# Theoretical Cost-Effectiveness of PCSK9 Inhibitors in Stroke Due to Intracranial Atherosclerosis

**DOI:** 10.1001/jamanetworkopen.2026.10707

**Published:** 2026-05-05

**Authors:** Caroline Kellogg, Elena Badillo Goicoechea, David D. Kim, Shadi Yaghi, Seemant Chaturvedi, Rachel Mehendale, Karen Orjuela, Tareq Kass-Hout, Rami Z. Morsi, Jacqueline Morales, Eesha Singh, Aditya Jhaveri, Shyam Prabhakaran, Matthew M. Smith, James R. Brorson, Samiksha Golani, Sofia Mazuera, Rachael Kang, Ali Eltatawy, Jessica Pillajo, James E. Siegler

**Affiliations:** 1 University of Chicago, Chicago, Illinois; 2 Department of Medicine, University of Chicago, Chicago, Illinois; 3 Department of Neurology, Lifespan Hospital, Providence, Rhode Island; 4 Department of Neurology, University of Maryland, Baltimore; 5 Department of Neurology, University of Chicago, Chicago, Illinois

## Abstract

**Importance:**

Given the high costs of treatment with proprotein convertase subtilisin/kexin type 9 inhibitors (PCSK9i) and the limited US Food and Drug Administration label for these treatments in patients with stroke due to atherosclerotic disease, it is unknown whether these agents are cost-effective for reducing recurrent stroke events.

**Objective:**

To estimate the theoretical cost-effectiveness of currently available PCSK9i for the prevention of recurrent stroke in patients with high-risk intracranial arterial stenosis.

**Design, Setting, and Participants:**

This economic evaluation was a post hoc trial-based cost-effectiveness analysis of the Stenting and Aggressive Medical Management for Preventing Recurrent Stroke in Intracranial Stenosis (SAMMPRIS) trial from a health care sector perspective. Adult patients with stroke due to intracranial arterial stenosis were included in SAMMPRIS and randomized to aggressive medical management with or without intracranial arterial stenting. Patients from the SAMMPRIS trial with complete covariate data were included in this secondary analysis. Data were analyzed from January to December 2025.

**Exposure:**

A base-case assumption of 32% relative risk reduction for stroke among PCSK9i based on its low-density lipoprotein–lowering effect.

**Main Outcomes and Measures:**

Based on trial-estimated transition probabilities, annual direct-to-consumer prices for 3 PCSK9i (alirocumab: $6600; evolocumab: $7200; and inclisiran: $7920) and estimated US-based stroke care costs in 2025 US dollars, a decision-analytic Markov cohort model was developed to estimate the cost-effectiveness of PCSK9i for reducing recurrent stroke over a 5-year time horizon, with a 7% annual drug discontinuation rate and a 3% discount rate for future costs and quality-adjusted life-year (QALYs). From 1000 Monte Carlo simulations, the probability of cost-effectiveness of 3 PCSK9i was estimated at a $120 000/QALY threshold. Sensitivity analyses evaluated at an alternative threshold ($50 000/QALY), treatment efficacy (20% to 50%), and stroke care costs (50% to 150% of base estimates). A patient perspective analysis reflecting patients’ out-of-pocket costs with insurance coverage was also conducted.

**Results:**

Of the 367 patients from the SAMMPRIS included in this study, 88 were Black (24.0%), 260 were White (70.8%), and 19 Asian, Native Hawaiian or Pacific Islander, more than 1 race, or other (5.2%), and the median (IQR) age at enrollment was 59 (52-69) years. At current direct-to-consumer prices, the probability of cost-effectiveness at a willingness-to-pay threshold of $120 000/QALY over a 5-year horizon was 58.6% (95% CI, 55.5%-61.6%) for alirocumab, 53.8% (95% CI, 50.7%-56.9%) for evolocumab, and 36.7% (95% CI, 33.8%-39.7%) for inclisiran.

**Conclusions and Relevance:**

This theoretical framework suggests that alirocumab and evolocumab were cost-effective for preventing recurrent stroke in patients with severe intracranial atherosclerosis, with all agents cost-effective under current cost-sharing programs offered by commercial and Medicare plans.

## Key Points

**Question:**

Given the high costs of treatment, are proprotein convertase subtilisin/kexin type 9 inhibitors (PCSK9i) cost-effective for patients with stroke due to intracranial atherosclerosis?

**Findings:**

This economic evaluation of 367 patients with recent strokes attributed to intracranial atherosclerotic stenosis used theoretical estimates of PCSK9i treatment effect and found that the probability of cost-effectiveness for reducing recurrent stroke at 5 years was 58.6% for alirocumab, 53.8% for evolocumab, and 36.7% for inclisiran.

**Meaning:**

These findings suggest that within this theoretical framework, alirocumab and evolocumab were cost-effective for preventing recurrent stroke at current direct-to-consumer annual prices of $6600 and $7200, respectively.

## Teaser

This economic evaluation estimates the theoretical cost-effectiveness of currently available proprotein convertase subtilisin/kexin type 9 inhibitors (PCSK9i) for the prevention of stroke in patients with high-risk intracranial arterial stenosis.

## Introduction

Dyslipidemia is a major risk factor for cardiovascular disease, which affects more than 523 million people globally and represents a leading cause of death.^1^ It encompasses any disorder that causes the disruption of lipid metabolism and is characterized by abnormal levels of fats in the blood, such as low-density lipoprotein (LDL). The most recent 2021 American Heart Association/American Stroke Association Guidelines recommend LDL lowering to a target level using high-intensity statins for the secondary prevention of ischemic stroke.^2^ However, there are cases where statin therapy alone cannot lower LDL levels to the recommended target, may be poorly tolerated, or lead to significant toxicity. Newer therapies, including proprotein convertase subtilisin/kexin type 9 inhibitors (PCSK9i), may help achieve target LDL levels for these patients.

While PCSK9i are effective at treating dyslipidemia, their cost can be prohibitive at the patient level or for the health care system more broadly. To date, there has not been a study evaluating the cost-effectiveness of PCSK9i use for the prevention of acute ischemic stroke in patients prescribed these therapies for an index ischemic stroke. This is largely due to the lack of clinical trials that have tested the benefit of these therapies in patients exclusively with a history of cerebral infarction. Because certain stroke populations are at a heightened risk of recurrent stroke despite optimal medical therapy and current LDL targets (particularly among patients with stroke due to intracranial atherosclerosis), many are increasingly prescribed PCSK9i to augment lipid control and reduce the risk of recurrent cerebrovascular events.^3^ Previously, we have shown that the theoretical use of these agents could reduce recurrent atherosclerotic events (the majority of which were recurrent ischemic strokes) by more than 50% with the use of PCSK9i when added to maximal medical therapy.^4^ In this study, we expanded upon this theoretical framework by exploring the cost-effectiveness of the theoretical use of these agents.

## Methods

Data from the Stenting and Aggressive Medical Management for Preventing Recurrent Stroke in Intracranial Stenosis (SAMMPRIS) trial were generously provided by the National Institute of Neurological Disorders and Stroke upon request of the corresponding author and can be obtained by other investigators upon such a request (NCT00576693). This economic evaluation followed the Consolidated Health Economic Evaluation Reporting Standards (CHEERS) reporting guideline.^5^ The SAMMPRIS trial protocol was approved by the institutional review boards (IRBs) of participating sites, US Food and Drug Administration, and Data and Safety Monitoring Board appointed by the National Institutes of Health. This secondary analysis of deidentified data was exempt from IRB review under the Common Rule.

### Statistical Analysis

In this post hoc cost-effectiveness analysis of patients with recent ischemic stroke due to high-grade intracranial atherostenosis (70% to 99%) enrolled in the SAMMPRIS trial, we evaluated the hypothetical addition of PCSK9i therapy to standard medical therapy (the intervention) compared with standard medical therapy alone (the comparator) for secondary stroke prevention in patients with severe symptomatic intracranial atherosclerosis (eTable 1 in Supplement 1). Although most costs associated with PCSK9i prescriptions are shared by insurance providers and available through cost-sharing programs, we chose to conduct the primary, base-case cost-effectiveness analysis from a health care sector perspective, with costs reflecting current US health care expenditures using publicly available direct-to-consumer prices as a proxy for net costs to the health care system. Secondary analyses included drug costs reported by the Federal Supply Schedule (FSS), as of December 2, 2025, and out-of-pocket costs incurred by US-based Medicare and commercial beneficiaries. The primary outcome was cost per quality-adjusted life-year (QALY) gained, evaluated at willingness-to-pay (WTP) thresholds of $50 000/QALY and $120 000/QALY, the latter representing the recently updated American Heart Association/American College of Cardiology recommendation.^6^

#### Model Structure and Simulation

We developed a decision-analytic Markov cohort model to estimate the cost-effectiveness of administering PCSK9i to a hypothetical patient population similar to that in SAMMPRIS, accounting for both medication and stroke care costs. We used a 5-year time horizon with a 3% annual discount rate for both costs and QALYs, consistent with US Panel on Cost-Effectiveness in Health and Medicine recommendations,^7^ and an annual drug discontinuation rate of 7%.^8,9^ A 5-year time horizon was selected owing to the imprecision of long-term associations between LDL reduction and recurrent stroke based on data from the SAMMPRIS trial, where the median (IQR) follow-up was 236 (131-506) days.

The model incorporated 3 mutually exclusive health states: stable poststroke, recurrent stroke, and death, with their corresponding transition probabilities. In each simulation, patients began in the stable poststroke state and could transition between states according to the annual probabilities of recurrent stroke (modified by PCSK9i treatment effect in the intervention group) and mortality (eTable 1 in Supplement 1). The model compared outcomes between the PCSK9i intervention group and the standard care comparator group, calculating incremental costs, incremental QALYs, and incremental cost-effectiveness ratios (ICERs) for each simulation iteration. Details about the probabilistic distributions for the model can be found in the eMethods in Supplement 1.

#### Theoretical Treatment Effectiveness

Direct experimental evidence for PCSK9i effects on recurrent stroke in patients with intracranial atherosclerosis is limited. Therefore, we used an evidence synthesis approach linking 2 established associations. We fitted a multivariable Cox model to estimate the risk of recurrent ischemic stroke according to LDL reduction in the SAMMPRIS population (eAppendix in Supplement 1). Second, we incorporated the mean (SD) effect of PCSK9i treatment on LDL reduction (60% [0.05%]) from a network meta-analysis of 48 randomized clinical trials evaluating PCSK9i added to statin therapy.^10,11^ We applied this LDL reduction to each patient in the SAMMPRIS cohort and used the fitted Cox model to project reductions in incidence rate for recurrent cerebral infarction using predictive margins. Because there was no significant interaction between LDL reduction and recurrent stroke risk across the 2 randomized patient groups in SAMMPRIS (stenting and then best medical management compared with the best medical management alone; *P* = .11), the 2 groups were combined for the present analysis. These methods yielded our base-case estimate of relative risk reduction for recurrent stroke with PCSK9i added to statin therapy compared with statin therapy alone. To account for the potential diminishing marginal benefits of PCSK9i on LDL in patients with already low LDL levels, the projected outcome was made logarithmically dependent on each patient’s baseline LDL level. Estimated differences in annualized incidence rates were calculated across the included cohort by contrasting the projected change in outcomes under PCSK9i vs no PCSK9i (using the original LDL changes observed in the SAMMPRIS data) for each individual in the sample, with all other factors left constant in the model.

#### Other Clinical Data

We populated the model with published epidemiologic data on stroke recurrence and mortality, with full details available in the eAppendix in Supplement 1. The baseline annual recurrent stroke rate of 14.9% was derived from the medically managed group of the SAMMPRIS trial (patients with 70%-99% stenosis).^12^ We incorporated the associated uncertainty (ie, SD = 0.008) to reflect interstudy variability. Background mortality (2% annually) and poststroke mortality (5% annually) were obtained from vital statistics and observational cohorts of patients with symptomatic intracranial stenosis.^12,13^ Health state utilities were assigned based on published preference studies: 0.9 for stable poststroke state (representing a modified Rankin Scale of 0-1)^14,15^ and 0.5 for recurrent stroke state,^16^ with uncertainty (ie, SD = 0.15) incorporated via β distributions in probabilistic analyses.

#### Costs and Resources

In the primary analysis, we evaluated 3 FDA-approved PCSK9i at current direct-to-consumer prices (2025 USD): alirocumab ($6600 annually), evolocumab ($7200 annually), and inclisiran ($10 800 for year 1, then $7200 annually). We incorporated a 20% coefficient of variation to reflect price uncertainty.

#### Uncertainty and Sensitivity Analyses

We conducted probabilistic sensitivity analyses using 1000 Monte Carlo simulations based on specified parameter values for probabilistic distributions to characterize parameter uncertainty (eTable 1 in Supplement 1). Probabilities of cost-effectiveness are described in proportions based on event rates out of 1000 simulations in which costs of PCSK9i treatment are exceeded by predicted 5-year health care costs. Monte Carlo sampling variability was estimated using Wilson confidence intervals to generate 95% CIs. To address the inherent uncertainty in our evidence synthesis approach, we also conducted scenario analyses to test alternative treatment effectiveness assumptions of 20% (conservative) and 50% (optimistic) relative risk reduction for recurrent stroke to acknowledge methodological uncertainty in combining the LDL-stroke risk association from SAMMPRIS with PCSK9i outcomes on LDL from external trials. We also tested alternative stroke care costs at 50% and 150% of the base-case estimates, and alternative PCSK9i costs of $3000 and $8000 annually. As a threshold analysis, we estimated the annualized cost of PCSK9i at which the probability of cost-effectiveness would exceed 50% at the $120 000/QALY WTP threshold. A cost-effectiveness probability of less than 50% was considered not cost-effective, 50% was considered the equipoise case, and more than 75% was considered a high probability of cost-effectiveness.

In a secondary analysis, we repeated all analyses from a patient perspective using out-of-pocket costs after insurance coverage. For Medicare and commercial insurance beneficiaries, we used $600 annually for alirocumab and evolocumab (reflecting typical copayment structures), and $2160 in year 1, followed by $1440 annually for inclisiran (reflecting 20% cost-sharing under typical Medicare Part B coverage). Lastly, we included an additional threshold analysis identifying the annual PCSK9i price at which 50%, 75%, and 90% probability of cost-effectiveness would be achieved at both the $50 000/QALY and $120 000/QALY WTP thresholds. This allowed the evaluation of the price-probability association across a full range of decision-relevant confidence levels.

All analyses were performed using R version 4.3.2 (R Project for Statistical Computing). Statistical significance was set at *P* < .05. Data were analyzed from January to December 2025.

## Results

**Figure 1.**
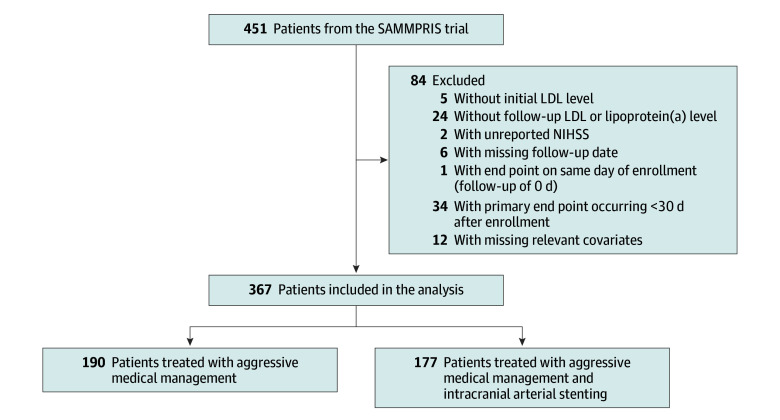
Study Flow Chart LDL, low-density lipoprotein; NIHSS, National Institutes of Health Stroke scale; SAMMPRIS, stenting and aggressive medical management for preventing recurrent stroke in intracranial stenosis.

From the 451 patients in the SAMMPRIS trial, which included patients with recent strokes attributed to intracranial atherosclerotic stenosis, 367 patients had complete covariate data and were included in this secondary analysis (Figure 1). Of the 367 patients included in this study, 88 were Black (24.0%), 260 were White (70.8%), and 19 Asian, Native Hawaiian or Pacific Islander, more than 1 race, or Other (5.2%), and the median (IQR) age at enrollment was 59 (52.0-69.0) years (Table 1).

**Table 1.** Baseline Patient Characteristics

Characteristic	Patients, No. (%) (N = 367)
Demographics	
Age at enrollment, median (IQR), y	59 (52-69)
Sex	
Female	133 (36.2)
Male	234 (63.8)
Ethnicity	
Hispanic or Latino	29 (7.9)
Non-Hispanic	338 (92.1)
Race	
Black	88 (24.0)
White	260 (70.8)
Other^a^	19 (5.2)
Clinical characteristics	
BMI, median (IQR)	29 (26.4-34)
NIHSS Total score, median (IQR)	1 (0-2)
Angiographic stenosis, median (IQR), %	75 (70-81)
Medical history	
Hypertension	330 (89.9)
Diabetes	149 (40.6)
Prior stroke	88 (24.0)
Prior myocardial infarction	49 (13.4)
Congestive heart failure	8 (2.2)
Active tobacco use	138 (37.6)
Statin use at enrollment	316 (86.1)
Baseline LDL cholesterol, median (IQR), mg/dL	91 (72.0 to 116)
LDL change from baseline to 30 d, median (IQR), mg/dL	−19 (−41 to −1)
Outcomes	
Primary end point event (stroke)	62 (16.9)
Time to primary end point, median (IQR), d	1026 (796 to 1244)

Abbreviations: BMI, body mass index (calculated as weight in kilograms divided by height in meters squared); LDL, low-density lipoprotein; NIHSS, National Institutes of Health Stroke scale.

SI conversion facter: To convert LDL from mg/dL to mmol/L, multiply by 0.0259.

^a^ Other includes Asian, Native Hawaiian or Pacific Islander, more than 1 race, or other.

**Figure 2.**
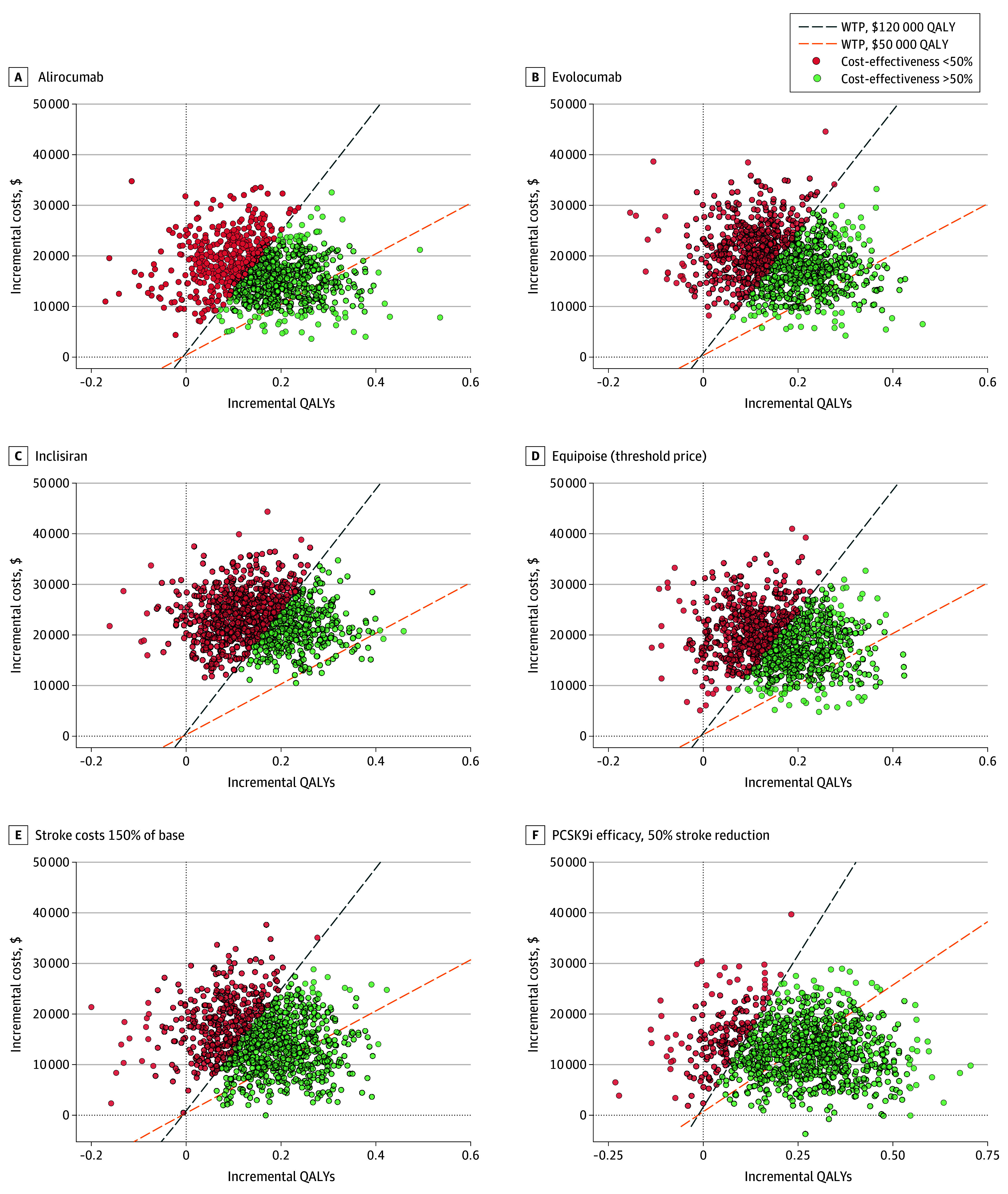
Scatterplot of Cost-Effectiveness Planes of Proprotein Convertase Subtilisin/Kexin 9 Inhibitor (PCSK9i) Cost-Effectiveness at Direct-to-Consumer Prices These cost-effectiveness planes illustrate simulations across given scenarios, with the y-axis corresponding to incremental estimated 5-year costs of care (in 2025 US dollars), and the x-axis corresponding to incremental quality-adjusted life-year (QALY) with treatment. Costs of each agent represent direct-to-consumer costs, excluding cost-sharing by insurance programs. Orange dashed lines correspond to a willingness-to-pay (WTP) of $50 000/QALY, and blue dashed lines correspond to a WTP of $120 000/QALY. In addition, simulations in green indicate cost-effectiveness of more than 50%, while those in red indicate cost-effectiveness of less than 50%. Simulations are shown for alirocumab (58.6% [95% CI, 55.5%-61.6%]) (A), evolocumab (53.8% [95% CI, 50.7%-56.9%) (B), inclisiran (36.7% [95% CI, 33.8%-39.7%]) (C), and equipoise case (threshold price) at which the treatment has a 50% probability of achieving cost-effectiveness (D). Also shown are sensitivity analyses for the equipoise case, in which costs of recurrent stroke are increased by 50% (E), and PCSK9i treatment effectiveness is increased (ie, 50% reduction) (F).

We projected a theoretical 32% reduction in relative stroke risk with PCSK9i use in combination with statins for our target patient population, based on the SAMMPRIS trial.^4^ This estimate, along with the rest of the input parameters, allowed us to build a base scenario Markov model to project the QALYs and cost-effectiveness. We found that PCSK9i in combination with statins generated an additional 0.16 (95% CI, 0.05-0.27) QALYs compared with statins alone (Table 2). When modeled for alirocumab, evolocumab, and inclisiran at their respective current direct-to-consumer prices, there was a probability of cost-effectiveness of 58.6% (95% CI, 55.5%-61.6%), 53.8% (95% CI, 50.7-56.9), and 36.7% (95% CI, 33.8%-39.7%), respectively, associated with these interventions at a WTP threshold of $120 000/QALY (Table 2 and Figure 2).

**Table 2.** Model Outcomes

Model input and outcome	Alirocumab	Evolocumab	Inclisiran	Threshold price^a^
**Outcomes**				
Costs, median (95% CI)				
Standard care, $	35 832 (30 530 to 41 901)	35 954 (30 664 to 41 748)	35 999 (30 720 to 41 662)	35 823 (30 421 to 41 523)
PCSK9i, $	57 308 (45 006 to 71 968)	60 211 (46 417 to 76 776)	63 476 (51 723 to 76 393)	57 351 (45 197 to 71 607)
Incremental, $	21 475 (9740 to 34 958)	24 257 (11 722 to 39 293)	27 478 (16 729 to 40 578)	21 528 (10 493 to 35 755)
QALYs, median (IQR)				
Standard care	3.30 (2.28 to 4.03)	3.32 (2.29 to 4.02)	3.31 (2.28 to 4.02)	3.33 (2.30 to 4.06)
PCSK9i	3.49 (2.41 to 4.11)	3.51 (2.34 to 4.14)	3.50 (2.39 to 4.10)	3.52 (2.38 to 4.15)
Incremental	0.19 (−0.01 to 0.40)	0.19 (0.00 to 0.40)	0.19 (−0.01 to 0.40)	0.19 (−0.01 to 0.40)
Cost-effectiveness				
ICER, median (95% CI)	108 192 (38 821 to 725 616)	126 915 (40 414 to 1 379 429)	147 572 (54 174 to 929 937)	115 339 (35 943 to 683 576)
Probability CE at $50 000/QALY, %	12.3 ( 10.4-14.5)	8.1 (6.6-10.0)	1.5 (0.9-2.5)	7.5 (6.0-9.3)
Probability CE at $120 000/QALY, %	58.6 (55.5-61.6)	53.8 (50.7-56.9)	36.7 (33.8-39.7)	53.8 (50.7-56.9)

Abbreviations: CE, cost-effective; ICER, incremental cost-effectiveness ratio; PCSK9i, proprotein convertase subtilisin/kexin type 9 inhibitor; QALY, quality-adjusted life-year.

^a^ Annual medication cost at which PCSK9i achieves an approximate 50% probability of cost-effectiveness at $120 000/QALY (equipoise threshold).

We further computed the annual cost of PCSK9i that would yield 50% probability (equipoise case scenario) that treatment would be cost-effective at a WTP threshold of $120 000/QALY (Table 2 and Figure 2). This amounted to $7000 per year over a 5-year horizon.

### Sensitivity Analyses

**Figure 3.**
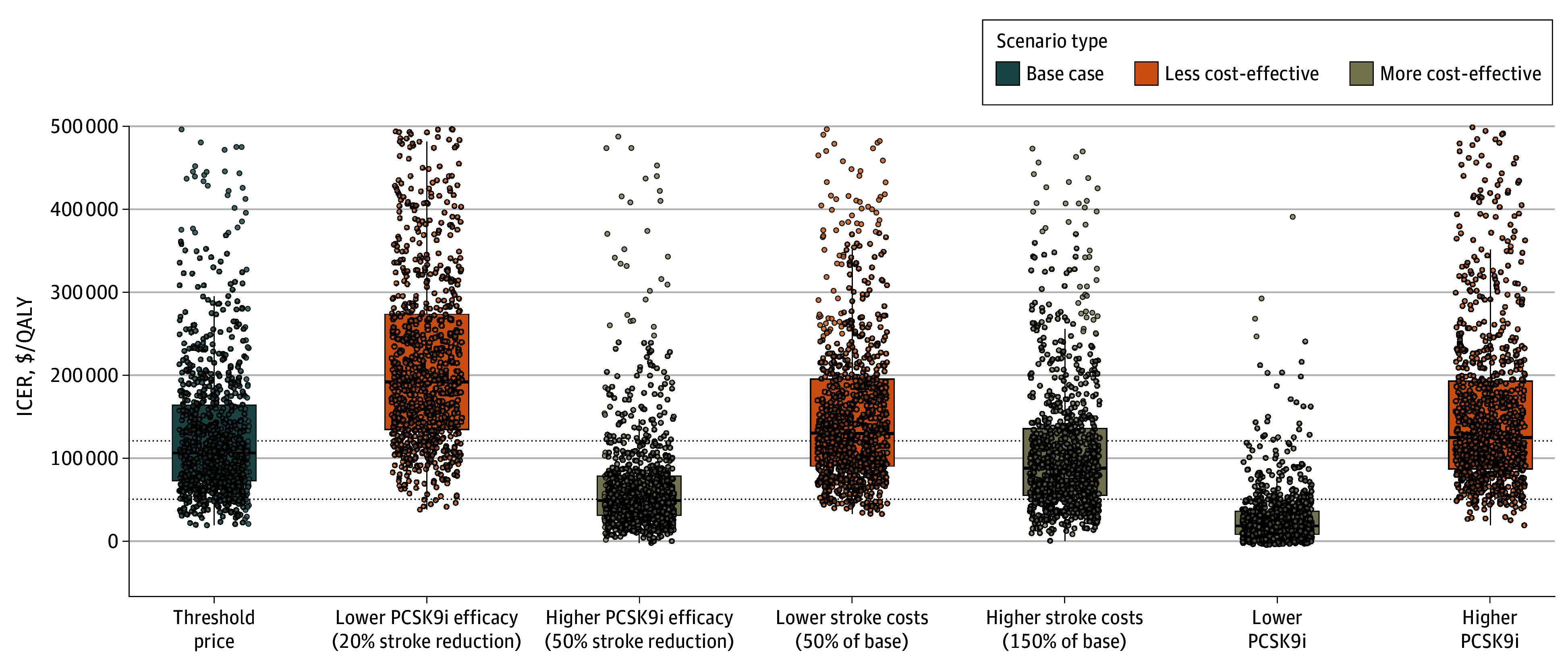
Box-and-Whisker Plots of Incremental Cost-Effectiveness Ratio (ICER): Baseline and Alternative Scenarios Over a 5-Year Horizon The y-axis represents the ICER in 2025 US dollars per quality-adjusted life year (QALY), and the x-axis represents each subgroup upon which simulations were run. Box-and-whisker plots correspond to median (IQR) ICER. Horizontal reference lines illustrate the differing willingness-to-pay thresholds for $120 000/QALY and $50 000/QALY. Dots indicate individual scenarios. PCSK9i indicates proprotein convertase subtilisin/kexin 9 inhibitor, and PCSK9i costs represent direct-to-consumer costs, not considering cost-sharing by insurance programs.

Considering the variability in our estimations, we performed sensitivity analyses of PCSK9i effectiveness, stroke costs, and PCSK9i costs. First, at a lower efficacy of treatment effect (20% stroke reduction), PCSK9i were not cost-effective at either a WTP threshold per QALY of $50 000 (alirocumab, 0.9% [95% CI, 0.5%-1.7%]; evolocumab, 0.4% [95% CI, 0.2%-1.0%]; inclisiran, 0% [0%-0.4%]) or $120 000 (alirocumab, 18.2% [95% CI, 15.9%-20.7%]; evolocumab, 14.0% [95% CI, 12.0%-16.3%]; inclisiran, 7.6% [95% CI, 6.1%-9.4%]); however, at 50% stroke reduction, PCSK9i became more cost-effective at a WTP threshold per QALY of $120 000 (alirocumab, 86.7% [95% CI, 84.5%-88.7%]; evolocumab, 83.3% [95% CI, 80.9%-85.5%]; inclisiran, 79.2% [76.6%-81.6%]) but still not at $50 000 (alirocumab, 57.0% [95% CI, 53.9%-60.0%]; evolocumab, 49.4% [95% CI, 46.3%-52.5%]; inclisiran, 29.5% [95% CI, 26.8%-32.4%]) (Figure 2). A similar outcome was observed for stroke care costs: lower care costs led to PCSK9i losing cost-effectiveness at both WTP thresholds at $120 000/QALY, cost-effectiveness for alirocumab was 47.6% [95% CI, 44.5%-50.7%], evolocumab was 41.2% [95% CI, 38.2%-44.3%], and inclisiran was 30.3% [95% CI, 27.5%-33.2%]), whereas higher care costs increased the probability of cost-effectiveness at the $120 000/QALY WTP alone (alirocumab, 71.1% [95% CI, 68.2%-73.8%]; evolocumab, 63.0% [95% CI, 60.0%-65.9%]; inclisiran, 48.4% [95% CI, 45.3%-51.5%]) (Figure 2). This pattern broke when examining PCSK9i costs. An annual direct-to-consumer cost of $3000 per year would not only increase the probability of PCSK9i being cost-effective at a WTP threshold of $120 000/QALY but also cause PCSK9i to become cost-effective at a WTP threshold of $50 000/QALY. A summary of the sensitivity analysis results was visualized in Figure 3.

When priced according to the FSS, there were modest improvements in cost-effectiveness of each agent at a WTP threshold per QALY of $120 000 (evolocumab, 65.2% [95% CI, 62.2%-68.1%]; alirocumab, 61.3% [95% CI, 58.2%-64.3%]; inclisiran, 45.2% [95% CI, 42.1%-48.3%]). Considering the out-of-pocket costs for most Medicare beneficiaries for evolucumab and alirocumab ($600 per year), the cost-effectiveness at a WTP threshold per QALY of $120 000 was 99.0% (95% CI, 98.2%-99.5%) and 99.4% (95% CI, 98.7%-99.7%) for evolocumab and alirocumab, respectively, and 97.3% (95% CI, 96.1%-98.1%) for inclisiran after 80% of the direct-to-consumer costs are covered. These agents remained cost-effective at a WTP threshold of $50 000 (99.8% [95% CI, 99.3%-99.9%] for evolocumab and alirocumab and 97.9% [95% CI, 96.8%-98.6%] for inclisiran). We further evaluated cost-effectiveness of PCSK9i under more stringent criteria, assuming a 75% probability and 90% probabilities of cost-effectiveness, which, respectively, yielded an annual PCSK9i equipoise price of approximately $3900 per year and $2000 per year (eTable 2 in Supplement 1).

## Discussion

In this economic evaluation, we evaluated the cost-effectiveness of PCSK9i for preventing recurrent ischemic stroke in patients with high-risk intracranial arterial stenosis using multiple cost estimates, including direct-to-consumer costs, FSS costs, and shared cost programs offered by the manufacturers. At their direct-to-consumer costs, alirocumab and evolocumab modestly exceeded the equipose threshold for cost-effectiveness at $6600 per year and $7200 per year, respectively, while inclisiran did not. In cost-sharing programs, the majority of agents surpassed the equipoise threshold for cost-effectiveness and remained cost-effective across a range of WTP thresholds and recurrent stroke risk levels (eFigure in Supplement 1). While the framework used in this model was based on theoretical treatment benefits extrapolating data from a clinical trial, there were no other high-quality data to source for an evaluation of the theoretical LDL-lowering effect of PCSK9i in patients with high-risk atherosclerotic intracranial vasculopathy. Strengths of this analysis included the multiple sensitivity analyses exploring a range of LDL-lowering effects, a range of costs faced by consumers, a range of WTP thresholds, and conventional modeling parameters, inclusive of standard discounting and known drug discontinuation rates for PCSK9i related to adverse effects. For patients with insurance, if most of the cost-sharing was covered by insurance through each individual agent’s copay program, the out-of-pocket costs to consumers were low, and each agent was cost-effective across multiple WTP thresholds.

These cost-effectiveness analyses were broadly consistent with prior literature for other patient populations and endpoints, which found that PCSK9i could be marginally cost-effective at a WTP threshold of $100 000/QALY when priced at $5459 annually.^17^ Unlike prior analyses of PCSK9i cost-effectiveness, the more apparent cost-effectiveness of alirocumab and evolocumab at current direct-to-consumer prices for the prevention of recurrent stroke was likely due to the considerably high risk of recurrent stroke in patients with intracranial atherostenosis, with high costs of subsequent disability and other health care expenses. More aggressive LDL-lowering with PCSK9i had a powerful theoretical, absolute benefit based on our prior analysis.^4^ Additionally, the American Heart Association recently updated the annual WTP threshold to $120 000, which provides greater leniency for frontloaded health care costs associated with PCSK9i.^6^

The findings of this study have important implications for the use of PCSK9i. The current FDA labels for these agents do not indicate them for use in patients with established cerebrovascular disease. However, for this subset of patients with persistently high LDL levels and markedly high risk of recurrent stroke, PCSK9i have been shown to significantly reduce the risk of recurrent stroke in a theoretical framework.^4^ Moreover, this outcome has been reported in randomized clinical trials for primary prevention of ischemic stroke,^3,18^ where the magnitude of clinical benefit is likely less than for secondary prevention when the rate of stroke recurrence is much higher.

There are several factors that could have led to the underestimation of the cost-effectiveness of PCSK9i in this population, including additional potential benefits of PCSK9i beyond stroke prevention and changes in the broader health care system. For example, PCSK9i could also reduce the risk of vascular events such as myocardial infarction, thereby increasing its cost-effectiveness at the same price. However, these event rates in relation to lower LDL levels after ischemic stroke have not been well defined, and we reported that a minority of recurrent vascular events in the SAMMPRIS trials were due to myocardial infarction (1 in 6 events).^4^ Furthermore, it is complex to estimate the costs associated with accumulated disability from myocardial infarction and symptomatic peripheral artery disease, so we chose not to include these cost savings in this model.

Additionally, LDL control with PCSK9i could also reduce vascular deaths. According to data from the prospective Oxford Vascular Study, the risk of death was more than twice as great as the risk of recurrent ischemic stroke in patients with intracranial atherostenosis greater than 50%.^13^ In the original SAMMPRIS results, a minority of patients experienced death as defined in the primary outcome (1.3% of the trial population, comprising 13% of the primary composite endpoint events in the trial).^4,19^ Therefore, if death and myocardial infarction were included in the outcome of this cost-effectiveness framework, acknowledging that lower LDL levels may reduce the risk of these events,^20^ it is possible that inclisiran might have achieved a cost-effectiveness probability of more than 50% (in addition to alirocumab and evolocumab), particularly for patients with higher baseline LDL levels.^21^ That said, the mortality benefit of PCSK9i has not been well established according to randomized clinical trial data.^21^ Due to the scarcity of these data in stroke populations and the small event rate in SAMMPRIS, we did not include death, vascular death, or other disabling atherosclerotic vascular events in our cost-effectiveness modeling. Therefore, future studies are called upon to explore the cost-effectiveness of these agents for the prevention of other atherosclerotic vascular events beyond stroke. Furthermore, our model captures the indirect mortality benefit of PCSK9i through prevention of recurrent stroke, which carries a 5% annual case fatality rate. However, potential direct outcomes of PCSK9i on nonstroke cardiovascular mortality or all-cause mortality were not modeled, as these benefits have not been conclusively established in randomized clinical trial data. To the extent that they are present, these added benefits would improve the cost-effectiveness of PCSK9i across all our scenarios.

### Limitations

This study has limitations. While this analysis provides a robust estimation of cost-effectiveness of currently available PCSK9i for preventing recurrent ischemic stroke in a high-risk patient population, this is only a theoretical framework that applies largely to a US population. Until high quality randomized clinical trials are conducted that provide estimates of clinical benefit in secondary stroke prevention with PCSK9i, we cannot know with certainty the magnitude of benefit of these therapies (inclusive of their pleiotropic effects, including atheroma volume reduction,^22^ lowering of lipoprotein levels,^23^ and other mechanisms). Furthermore, the population from whom many clinical estimates were derived (SAMMPRIS trial participants) had minimal baseline symptoms with a median National Institutes of Health Stroke scale of 1. This may overestimate the baseline utility value when compared with other patients with stroke related to intracranial atherosclerosis. However, a lower utility value for a baseline or stable stroke state may increase the incremental QALY benefit of PCSK9i by amplifying the utility decrement from recurrent stroke, potentially augment its cost-effectiveness.

In this study, we also estimated the theoretical cost-effectiveness of PCSK9i use for the prevention of recurrent ischemic stroke. There are other known benefits of PCSK9i that could improve QALYs that are associated with the prevention of ischemic heart disease, reduction in stroke-related disability, increase in work and life productivity, indirect loss of productivity from family members who serve as caregivers, and fewer hospital readmissions,^24^ which are difficult to capture in a cost-effectiveness analyses. These indirect and pleiotropic benefits of PCSK9i, such as reduction of plaque volume and improvement in arterial luminal stenosis, are beyond the scope of the present study. The study was also limited by the small sample size.

## Conclusions

 The findings of this economic evaluation offer insight into the cost-effective utilization of these agents for the prevention of recurrent cerebrovascular events in patients with symptomatic intracranial arterial stenosis. The risk of recurrent stroke, and subsequent disability and mortality, was high in this population and continued to grow with time. Despite the seemingly high direct-to-consumer prices and, for many patients, cost-sharing prices for PCSK9i, which may be cost-prohibitive, these agents may ultimately lower the costs of subsequent health care-related expenses while also reducing the risk of neurological disability.

## Notes

Supplement 1. **eAppendix.** SAMMPRIS Trial Details
eMethods.
**eTable 1.** Baseline Model Inputs and Parameters **eTable 2.** Equipoise PCSK9i Prices at Different Cost-Effectiveness Probabilities **eFigure.** Patient Costs With Available Cost-Sharing Programs
eReferences.

Supplement 2.
Data Sharing Statement

## References

[1] Nedkoff L, Briffa T, Zemedikun D, Herrington S, Wright FL. Global trends in atherosclerotic cardiovascular disease. Clin Ther. 2023;45(11):1087-1091. doi:10.1016/j.clinthera.2023.09.020 37914585

[2] Kleindorfer DO, Towfighi A, Chaturvedi S, . 2021 Guideline for the prevention of stroke in patients with stroke and transient ischemic attack: a guideline from the American Heart Association/American Stroke Association. Stroke. 2021;52(7):e364-e467. doi:10.1161/STR.0000000000000375 34024117

[3] Giugliano RP, Pedersen TR, Saver JL, ; FOURIER Investigators. Stroke prevention with the PCSK9 (Proprotein Convertase Subtilisin-Kexin Type 9) inhibitor evolocumab added to statin in high-risk patients with stable atherosclerosis. Stroke. 2020;51(5):1546-1554. doi:10.1161/STROKEAHA.119.027759 32312223

[4] Siegler JE, Badillo Goicoechea E, Yaghi S, . Estimated theoretical benefit of aggressive ldl lowering in patients with symptomatic intracranial atherosclerosis. Neurology. 2025;105(1):e213768. doi:10.1212/WNL.0000000000213768 40446174

[5] Husereau D, Drummond M, Augustovski F, ; CHEERS 2022 ISPOR Good Research Practices Task Force. Consolidated Health Economic Evaluation Reporting Standards 2022 (CHEERS 2022) statement: updated reporting guidance for health economic evaluations. Value Health. 2022;25(1):3-9. doi:10.1016/j.jval.2021.11.1351 35031096

[6] Kazi DS, Abdullah AR, Arnold SV, ; Writing Committee Members. 2025 AHA/ACC statement on cost/value methodology in clinical practice guidelines (update from 2014 statement): a report of the American College of Cardiology/American Heart Association Joint Committee on Clinical Practice Guidelines. Circulation. 2025;152(18):e332-e358. doi:10.1161/CIR.0000000000001377 40997143

[7] Weinstein MC, Siegel JE, Gold MR, Kamlet MS, Russell LB. Recommendations of the panel on cost-effectiveness in health and medicine. JAMA. 1996;276(15):1253-1258. doi:10.1001/jama.1996.03540150055031 8849754

[8] Koren MJ, Sabatine MS, Giugliano RP, . Long-term efficacy and safety of evolocumab in patients with hypercholesterolemia. J Am Coll Cardiol. 2019;74(17):2132-2146. doi:10.1016/j.jacc.2019.08.1024 31648705

[9] Koren MJ, Sabatine MS, Giugliano RP, . Long-term low-density lipoprotein cholesterol-lowering efficacy, persistence, and safety of evolocumab in treatment of hypercholesterolemia: results up to 4 years from the open-label OSLER-1 extension study. JAMA Cardiol. 2017;2(6):598-607. doi:10.1001/jamacardio.2017.0747 28291870 PMC5815032

[10] Sabatine MS, Giugliano RP, Keech AC, ; FOURIER Steering Committee and Investigators. Evolocumab and clinical outcomes in patients with cardiovascular disease. N Engl J Med. 2017;376(18):1713-1722. doi:10.1056/NEJMoa1615664 28304224

[11] Ray KK, Ginsberg HN, Davidson MH, . Reductions in atherogenic lipids and major cardiovascular events: a pooled analysis of 10 ODYSSEY trials comparing alirocumab with control. Circulation. 2016;134(24):1931-1943. doi:10.1161/CIRCULATIONAHA.116.024604 27777279 PMC5147039

[12] Derdeyn CP, Chimowitz MI, Lynn MJ, ; Stenting and Aggressive Medical Management for Preventing Recurrent Stroke in Intracranial Stenosis Trial Investigators. Aggressive medical treatment with or without stenting in high-risk patients with intracranial artery stenosis (SAMMPRIS): the final results of a randomised trial. Lancet. 2014;383(9914):333-341. doi:10.1016/S0140-6736(13)62038-3 24168957 PMC3971471

[13] Hurford R, Wolters FJ, Li L, Lau KK, Küker W, Rothwell PM; Oxford Vascular Study Phenotyped Cohort. Prevalence, predictors, and prognosis of symptomatic intracranial stenosis in patients with transient ischaemic attack or minor stroke: a population-based cohort study. Lancet Neurol. 2020;19(5):413-421. doi:10.1016/S1474-4422(20)30079-X 32333899 PMC7116132

[14] Hong KS, Saver JL. Quantifying the value of stroke disability outcomes: WHO global burden of disease project disability weights for each level of the modified Rankin Scale. Stroke. 2009;40(12):3828-3833. doi:10.1161/STROKEAHA.109.561365 19797698 PMC2788070

[15] van Exel NJA, Scholte op Reimer WJ, Koopmanschap MA. Assessment of poststroke quality of life in cost-effectiveness studies: the usefulness of the Barthel Index and the EuroQoL-5D. Qual Life Res. 2004;13(2):427-433. doi:10.1023/B:QURE.0000018496.02968.50 15085915

[16] Tengs TO, Lin TH. A meta-analysis of quality-of-life estimates for stroke. Pharmacoeconomics. 2003;21(3):191-200. doi:10.2165/00019053-200321030-00004 12558469

[17] Arrieta A, Hong JC, Khera R, Virani SS, Krumholz HM, Nasir K. Updated cost-effectiveness assessments of PCSK9 inhibitors from the perspectives of the health system and private payers: insights derived from the FOURIER trial. JAMA Cardiol. 2017;2(12):1369-1374. doi:10.1001/jamacardio.2017.3655 29049467 PMC5814995

[18] Jukema JW, Zijlstra LE, Bhatt DL, ; ODYSSEY OUTCOMES Investigators. Effect of alirocumab on stroke in ODYSSEY Outcomes. Circulation. 2019;140(25):2054-2062. doi:10.1161/CIRCULATIONAHA.119.043826 31707788 PMC6919220

[19] Chimowitz MI, Lynn MJ, Derdeyn CP, ; SAMMPRIS Trial Investigators. Stenting versus aggressive medical therapy for intracranial arterial stenosis. N Engl J Med. 2011;365(11):993-1003. doi:10.1056/NEJMoa1105335 21899409 PMC3552515

[20] Steg PG, Szarek M, Bhatt DL, . Effect of alirocumab on mortality after acute coronary syndromes. Circulation. 2019;140(2):103-112. doi:10.1161/CIRCULATIONAHA.118.038840 31117810 PMC6661243

[21] Karatasakis A, Danek BA, Karacsonyi J, . Effect of PCSK9 inhibitors on clinical outcomes in patients with hypercholesterolemia: a meta-analysis of 35 randomized controlled trials. J Am Heart Assoc. 2017;6(12):e006910. doi:10.1161/JAHA.117.006910 29223954 PMC5779013

[22] Nicholls SJ, Puri R, Anderson T, . Effect of evolocumab on progression of coronary disease in statin-treated patients: the Glagov randomized clinical trial. JAMA. 2016;316(22):2373-2384. doi:10.1001/jama.2016.16951 27846344

[23] Szarek M, Bittner VA, Aylward P, ; ODYSSEY OUTCOMES Investigators. Lipoprotein(a) lowering by alirocumab reduces the total burden of cardiovascular events independent of low-density lipoprotein cholesterol lowering: ODYSSEY Outcomes trial. Eur Heart J. 2020;41(44):4245-4255. doi:10.1093/eurheartj/ehaa649 33051646 PMC7724642

[24] Szarek M, Steg PG, DiCenso D, . Alirocumab Reduces Total Hospitalizations and Increases Days Alive and Out of Hospital in the ODYSSEY Outcomes trial. Circ Cardiovasc Qual Outcomes. 2019;12(11):e005858. doi:10.1161/CIRCOUTCOMES.119.005858 31707826

