# Supplementary material for: Theoretical Cost-Effectiveness of PCSK9 Inhibitors in Stroke Due to Intracranial Atherosclerosis
Source: JAMA Netw Open. 2026 May 5;9(5):e2610707. doi: 10.1001/jamanetworkopen.2026.10707 (PMC13147199; doi:10.1001/jamanetworkopen.2026.10707)
Supplement: Supplement 1. — eAppendix. SAMMPRIS Trial Details eMethods. eTable 1. Baseline Model Inputs and Parameters eTable 2. Equipoise PCSK9i Prices at Different Cost-Effectiveness Probabilities eFigure. Patient Costs With Available Cost-Sharing Programs eReferences. [file jamanetwopen-e2610707-s001.pdf]

Supplemental Online Content

Kellogg C, Badillo Goicoechea E, Kim DD, et al. Theoretical cost-effectiveness of PCSK9 inhibitors in stroke due to intracranial atherosclerosis. *JAMA Netw Open*. 2026;9(5):e2610707.
doi:10.1001/jamanetworkopen.2026.10707

eAppendix. SAMMPRIS Trial Details
eMethods.
eTable 1. Baseline Model Inputs and Parameters
eTable 2. Equipoise PCSK9i Prices at Different Cost-Effectiveness Probabilities
eFigure. Cost-Effectiveness of PCSK9i With Cost-Sharing
eReferences.

This supplemental material has been provided by the authors to give readers additional information about their work.

© 2026 Kellogg C et al. *JAMA Network Open*.

eAppendix. SAMMPRIS Trial Details

SAMMPRIS Trial Overview and Randomization

The SAMMPRIS trial was a randomized, controlled, multicenter trial conducted at 50 sites in the United States. It enrolled patients with acute ischemic stroke between 2008 and 2011 and was designed to test the benefit of treating atherosclerotic patients with percutaneous transluminal angioplasty and stenting (PTAS) plus aggressive medical therapy versus aggressive medical therapy alone.[1] In SAMMPRIS, aggressive medical therapy was defined as dual antiplatelet therapy combined with management of both primary and secondary atherosclerotic vascular risk factors, which include diabetes, dyslipidemia, and excess body weight. To be eligible for the study, patients must have experienced a transient ischemic attack (TIA) or acute ischemic stroke caused by 70-99% stenosis of a major intracranial artery within 30 days of enrollment ("study entry"). Demographic patient data, such as race and Hispanic ethnicity were self-reported and captured by study investigators and team members.

Upon enrollment in SAMMPRIS, patients were managed aggressively for underlying vascular risk factors, including treatment with a high-intensity statin (rosuvastatin) to a target LDL of <70 mg/dL, antihypertensive agent(s) to achieve a goal systolic blood pressure of <140 mmHg (<130 mmHg for patients with diabetes), and additional diet and lifestyle modifications as described in the published trial protocol.[2] Following enrollment, patients were randomized to PTAS or to no stenting, with aggressive medical therapy continued for all included trial participants.

Primary and Secondary Aims

The primary aim of the SAMMPRIS trial was to determine whether PTAS with aggressive medical management was superior to aggressive medical management alone for the prevention of:

- Any stroke or death within 30 days of study entry
- Any stroke or death within 30 days of PTAS
- Ischemic stroke in the territory of the symptomatic artery from day 31 after study entry to completion of phase I of the trial

The secondary aim of the SAMMPRIS trial was to determine the long-term safety of PTAS for patients with symptomatic intracranial atherosclerosis stenosis.

Monitoring and follow-up during the SAMMPRIS Trial

After enrollment, patients were formally evaluated at study entry, at four days, at 30 days, and every 4 months until the last patient enrolled was followed for 1 year. The only exception to this is if a primary endpoint occurs; then, a patient would be evaluated 90 days after. To find more detailed information on the patient population and inclusion criteria, please refer to SAMMPRIS itself and this post hoc analysis.[2,3]

© 2026 Kellogg C et al. *JAMA Network Open*.

eMethods.

Recurrent Stroke Risk

Using individual patient data from SAMMPRIS (n=367 with complete covariate data), we fitted a multivariable Cox proportional hazards model with recurrent ischemic stroke as the outcome and relative change in LDL from baseline to day 30 as the primary exposure. The model adjusted for age, sex, body mass index, race, Hispanic ethnicity, hypertension, diabetes, myocardial infarction, congestive heart failure, statin use at enrollment, tobacco use, baseline National Institutes of Health Stroke Scale (NIHSS) score, and baseline LDL level. Proportional hazards assumptions were verified using Schoenfeld residuals. Ischemic stroke included clinical and radiographic evidence of cerebral infarction, excluding transient ischemic attacks. The effect estimates from this model were nearly identical to the effect estimates of a sensitivity analysis restricted to 6 covariates to avoid overfitting (covariates pre-specified as LDL improvement, age, sex, baseline NIHSS, statin use at enrollment, and baseline LDL level). This analysis has been described in detail previously.[3]

Health Care Costs

Acute stroke hospitalization costs ($24,654) and longitudinal stroke care costs were obtained from recent US-based cost-effectiveness studies.[4] We modeled year 1 post-stroke costs ($27,647), years 2-3 costs ($32,579 total over two years), and chronic stroke care costs for years 4-5 ($12,000 annually). All costs were adjusted to 2025 USD using the medical care component of the Consumer Price Index. We used beta and gamma distributions to model cost uncertainty with coefficients of variation ranging from 15-25%.

Parametric distributions

The primary model parameter derived from the trial data is the relative risk reduction for recurrent stroke with PCSK9i (base case: 32%, sampled as RR = 0.68, SD = 0.05 from a truncated normal distribution). This parameter was derived from a multivariable Cox model fitted to the SAMMPRIS data, which estimated the relationship between LDL reduction and recurrent stroke risk. The SD of 0.05 on the relative risk reflects the uncertainty in this estimate and was selected to capture the plausible range of treatment effects given the sample size and event rate.

We chose parametric distributions over bootstrap for two reasons. First, our model applies the treatment effect as a single relative risk modifier to the transition probability in each Markov cycle, making a parametric distribution on the relative risk a natural and computationally efficient approach. Second, the treatment effect is not directly observed in the trial but rather synthesized from two separate relationships (LDL-stroke risk from SAMMPRIS and LDL reduction from PCSK9i trials), making standard bootstrap of the trial data less directly applicable.

Uncertainty in the Cox model coefficients is an important contributor to overall parameter uncertainty, and our SD = 0.05 was chosen conservatively to encompass a wide range of treatment effects (approximately 18%-46% RRR at ±2 SD). We have also tested alternative treatment effectiveness assumptions (20% and 50% RRR) in our scenario analyses, which effectively bracket a much wider range than the parametric uncertainty alone.

© 2026 Kellogg C et al. *JAMA Network Open*.

eTable 1. Baseline Model Inputs and Parameters

Model input and parameter	Alirocumab	Evolocumab	Inclisiran	Threshold price[a]	Value	Source
Annual net PCSK9i cost (based on direct-to-consumer prices)[a]	$6600	$7200	$7920	$7000	NA	NA
PCSK9i cost SD (20%)	$1320	$1440	$1584	$1340	NA	NA
Time horizon, y	5	5	5	5	NA	NA
Discount rate, %	3	3	3	3	NA	NA
Annual drug discontinuation rate, %	7	7	7	7	NA	NA
Stroke hospitalization cost, $	24 654	24 654	24 654	24 654	NA	NA
Minimum plausible stroke cost, $	15 000	15 000	15 000	15 000	NA	NA
Maximum plausible stroke cost, $	45 000	45 000	45 000	45 000	NA	NA
Health state utilities						
Stable poststroke (mRS 0-1), mean (SD)	0.90 (0.15)	0.90 (0.15)	0.90 (0.15)	0.90 (0.15)	NA	NA
Recurrent stroke	0.50 (0.15)	0.50 (0.15)	0.50 (0.15)	0.50 (0.15)	NA	NA
Cost parameters						
Cost of stroke hospitalization	NA	NA	NA	NA	$24,654	Yousufuddin et al., 2020
Cost of stroke-related care after one year, including hospitalization	NA	NA	NA	NA	$27,647	Johnson et al., 2016
Quality of life parameters						
Stable post-stroke utility	NA	NA	NA	NA	0.9	Hong et al., 2009; van Exel et al., 2004
Recurrent stroke utility	NA	NA	NA	NA	0.5	Tengs et al., 2003
Mortality parameters						
Background mortality	NA	NA	NA	NA	2%	Derdeyn et al., 2014; Hurford

© 2026 Kellogg C et al. *JAMA Network Open.*

						et al., 2020
Poststroke mortality	NA	NA	NA	NA	5%	Derdeyn et al., 2014; Hurford et al., 2020

[a]All cost parameters have been adjusted to 2025 dollars with the BLS inflation calculator.

Yousufuddin M, Moriarty JP, Lackore KA, et al. Initial and subsequent 3-year cost after hospitalization for first acute ischemic stroke and intracerebral hemorrhage. J Neurol Sci. 419:117181. doi: 10.1016/j.jns.2020.117181.

Derdeyn CP, Chimowitz MI, Lynn MJ, et al. Aggressive medical treatment with or without stenting in high-risk patients with intracranial artery stenosis (SAMMPRIS): the final results of a randomised trial. Lancet. 2014;383(9914):333-341. doi:10.1016/S0140-6736(13)62038-3.

Tengs TO and Lin TH. A meta-analysis of quality-of-life estimates for stroke. Pharmacoeconomics. 2003;21(3): 191-200. doi:10.2165/00019053-200321030-00004.

Hurford R, Wolters FJ, Li L, et al. Prevalence, predictors, and prognosis of symptomatic intracranial stenosis in patients with transient ischaemic attack or minor stroke: a population-based cohort study. Lancet. 2020;19(5):413-421. doi: 10.1016/S1474-4422(20)30079-X.

Hong KS, Saver JL. Quantifying the value of stroke disability outcomes: WHO global burden of disease project disability weights for each level of the modified Rankin Scale. Stroke. 2009;40(12):3828-33. doi: 10.1161/STROKEAHA.109.561365.

van Exel NJA, Scholte op Reimer WJM, Koomanschap MA. Assessment of post-stroke quality of life in cost-effectiveness studies: the usefulness of the Barthel Index and the EuroQoL-5D. Qual Life Res. 2004;13(2):427-433. doi:10.1023/B:QURE.0000018496.02968.50.

© 2026 Kellogg C et al. *JAMA Network Open*.

eTable 2. Equipoise PCSK9i Prices at Different Cost-Effectiveness Probabilities

WTP Threshold	CE Probability	Equipoise Price
$50,000/QALY	50%	$5,500/year
$50,000/QALY	75%	$3,200/year
$50,000/QALY	90%	$1,800/year
**$120,000/QALY**	**50%**	**$6,900/year**
$120,000/QALY	75%	$3,900/year
$120,000/QALY	90%	$2,000/year

In this table, the acronyms denote: proprotein convertase subtilisin/kexin 9 inhibitor (PCSK9i), willingness-to-pay (WTP), cost-effectiveness (CE), and quality-adjusted life-year (QALY).

© 2026 Kellogg C et al. *JAMA Network Open*.

eFigure. Cost-Effectiveness of PCSK9i With Cost-Sharing

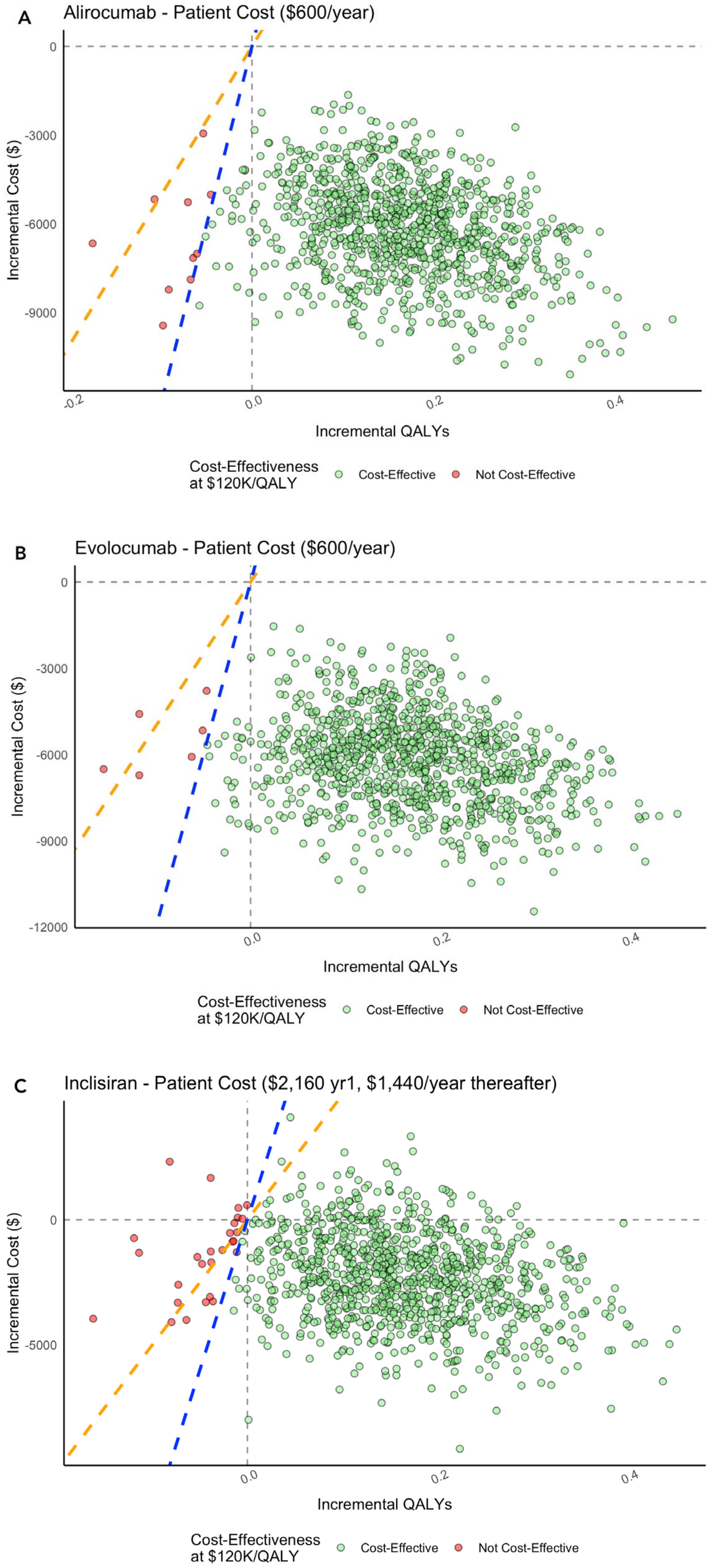

© 2026 Kellogg C et al. *JAMA Network Open*.

Cost-effectiveness planes illustrate simulations across given scenarios, with the y-axis corresponding to incremental estimated 5-year costs of care (in 2025 US dollars) and x-axis corresponding to incremental QALY with treatment. Costs represent common scenario costs of each PCSK9i with cost-sharing absorbed by commercial insurance providers. Orange dashed lines correspond to a WTP of $120,000/QALY, and blue dashed lines correspond to a WTP of $50,000/QALY. In addition, simulations in green indicate cost-effectiveness >50%, while those in red indicate cost-effectiveness <50%. Simulations shown for (A) alirocumab, (B) evolocumab, and (C) inclisiran.

© 2026 Kellogg C et al. *JAMA Network Open*.

eReferences

1. Chimowitz MI, Lynn MJ, Derdeyn CP, et al. Stenting versus aggressive medical therapy for intracranial arterial stenosis. *N Engl J Med*. 2011;365(11):993-1003. doi:10.1056/NEJMoa1105335

2. Chimowitz MI, Lynn MJ, Turan TN, et al. Design of the stenting and aggressive medical management for preventing recurrent stroke in intracranial stenosis trial. *J Stroke Cerebrovasc Dis*. 2011;20(4):357-368. doi:10.1016/j.jstrokecerebrovasdis.2011.05.001

3. Siegler JE, Badillo Goicoechea E, Yaghi S, et al. Estimated Theoretical Benefit of Aggressive LDL Lowering in Patients With Symptomatic Intracranial Atherosclerosis. *Neurology*. 2025;105(1):e213768. doi:10.1212/WNL.0000000000213768

4. Wechsler PM, Pandya A, Parikh NS, et al. Cost-Effectiveness of Increased Use of Dual Antiplatelet Therapy After High-Risk Transient Ischemic Attack or Minor Stroke. *JAHA*. 2024;13(7):e032808. doi:10.1161/JAHA.123.032808

© 2026 Kellogg C et al. *JAMA Network Open*.
