# Supplementary material for: Theoretical Cost-Effectiveness of PCSK9 Inhibitors in Stroke Due to Intracranial Atherosclerosis
Source: JAMA Netw Open. 2026 May 5;9(5):e2610707. doi: 10.1001/jamanetworkopen.2026.10707 (PMC13147199; doi:10.1001/jamanetworkopen.2026.10707)
Supplement: Supplement 2. — Data Sharing Statement [file jamanetwopen-e2610707-s002.pdf]

Data Sharing Statement

Kellogg. Theoretical Cost-Effectiveness of PCSK9 Inhibitors in Stroke Due to Intracranial Atherosclerosis. *JAMA Netw Open*. Published May 05, 2026.
doi:10.1001/jamanetworkopen.2026.10707

Data
Data available: Yes
Data types: Deidentified participant data
How to access data: Deidentified data from the SAMMPRIS trial can be requested from the NINDS.
When available: With publication

Supporting Documents
Document types: None

Additional Information
Who can access the data: Deidentified data from the SAMMPRIS trial can be requested from the NINDS.
Types of analyses: Analyses of these data will be made available upon reasonable request of the corresponding author.
Mechanisms of data availability: Deidentified data from the SAMMPRIS trial can be requested from the NINDS following application submission.
